# Generalized spatial coherence reconstruction for photoacoustic computed tomography

**DOI:** 10.1117/1.JBO.26.4.046002

**Published:** 2021-04-20

**Authors:** Jorge Tordera Mora, Xiaohua Feng, Nikhila Nyayapathi, Jun Xia, Liang Gao

**Affiliations:** a University of California Los Angeles, Samueli School of Engineering, Department of Bioengineering, California, United States; b University at Buffalo, School of Engineering and Applied Sciences, Department of Biomedical Engineering, Buffalo, New York, United States

**Keywords:** spatial coherence, photoacoustic tomography, beamformer

## Abstract

**Significance:** Coherence, a fundamental property of waves and fields, plays a key role in photoacoustic image reconstruction. Previously, techniques such as short-lag spatial coherence (SLSC) and filtered delay, multiply, and sum (FDMAS) have utilized spatial coherence to improve the reconstructed resolution and contrast with respect to delay-and-sum (DAS). While SLSC uses spatial coherence directly as the imaging contrast, FDMAS employs spatial coherence implicitly. Despite being more robust against noise, both techniques have their own drawbacks: SLSC does not preserve a relative signal magnitude, and FDMAS shows a reduced contrast-to-noise ratio.

**Aim:** To overcome these limitations, our aim is to develop a beamforming algorithm—generalized spatial coherence (GSC)—that unifies SLSC and FDMAS into a single equation and outperforms both beamformers.

**Approach:** We demonstrated the application of GSC in photoacoustic computed tomography (PACT) through simulation and experiments and compared it to previous beamformers: DAS, FDMAS, and SLSC.

**Results:** GSC outperforms the imaging metrics of previous state-of-the-art coherence-based beamformers in both simulation and experiments.

**Conclusions:** GSC is an innovative reconstruction algorithm for PACT, which combines the strengths of FDMAS and SLSC expanding PACT’s applications.

## 1 Introduction

Photoacoustic tomography (PAT) is a fast-growing, hybrid biomedical imaging technique. The combination of optical contrast and acoustic resolution endows PAT the capability to achieve label-free, high-resolution, structural, and functional imaging up to several centimeters deep in scattering media.^1^^,^^2^ In PAT, the sample is illuminated by a pulsed or intensity-modulated light source. The endogenous tissue components such as hemoglobin or melanin absorb light, raise their temperature a few millikelvin, and ultimately produce ultrasonic waves via thermal expansion.^3^ Among all PAT implementations, photoacoustic computed tomography (PACT) provides the most significant imaging depth and holds greatest promise for clinical translation. In a typical PACT system, the signals emitted from the sample are simultaneously collected by an array of transducers positioned at different locations and geometries such as spherical, circular, and linear.^4^^,^^5^ To reconstruct the image from these multi-sensor data, researchers have developed a wide range of reconstruction algorithms: backprojection (delay and sum)^6^ time-reversal,^7^ f-k migration,^8^^,^^9^ adaptive minimum variance,^10^^,^^11^ and deep learning-based approaches.^12^^,^^13^ However, these methods generally show suboptimal robustness to noise, which can easily overwhelm signals stemming from deep tissue regions where the light fluence is low, and the signals suffer from more substantial attenuation.

To abate noise, ultrasound imaging has long exploited spatial coherence by devising various coherence factors, such as phase coherence and sign coherence,^14^ to weight the output of the delay-and-sum (DAS) beamformer. As the side lobes and noises have low coherence, they can be effectively weighted down by coherence factors, thereby improving imaging resolution and contrast. Owing to the similarity of ultrasound and PACT reconstruction, these coherence factors have also been translated to PACT; despite that, a rigorous theory such as the van Cittert–Zernike (VCZ) theorem in ultrasound imaging was not established for photoacoustic imaging until recently.^15^ This is largely because the idea of exploiting spatial coherence between signals recorded at different sensors to discriminate against random noises is general. The recent linkage of the spatial coherence of photoacoustic signals to the VCZ theorem further solidifies its theoretical basis. Recently, instead of devising various coherence factors, better imaging performances have been achieved by either modifying the DAS beamformer to incorporate spatial coherence implicitly, as in filtered-delay-multiply and sum (FDMAS) beamformer,^16^^,^^17^ or more unconventionally, using directly spatial coherence as the imaging contrast, as in short-lag spatial coherence (SLSC).^18^ Both FDMAS and SLSC have enabled state-of-the-art image reconstruction with noisy data in ultrasound and photoacoustic imaging^19^^–^^22^ and have been further modified or combined with each other.^23^^–^^28^

Nevertheless, both beamformers still have drawbacks that limit their applicability. By computing normalized coherence values between the signals from different transducer elements as the imaging contrast, SLSC discards the signal magnitude information, preventing quantitative imaging such as oxygen saturation measurement in PACT. Unlike the SLSC beamformer, FDMAS relies on spatial coherence more implicitly: it deviates from DAS by adding a multiplication step between the delayed signals, which is essentially a correlation process that incorporates signal coherence. F-DMAS bolsters the imaging contrast without losing the signal magnitude. Yet, it suffers from a reduced contrast-to-noise ratio (CNR) for reasons that are not well understood.^29^

We present herein the generalized spatial coherence (GSC) beamformer, a novel reconstruction technique that offers state-of-the-art imaging contrast, CNR, and signal-to-noise ratio (SNR) for PACT. The GSC beamformer unifies SLSC and FDMAS into the same mathematical equation and, as such, sheds new insights on F-DMAS and SLSC. As an example, we explained the reason for the reduction of CNR in F-DMAS and the way that GSC rectifies it. The remainder of the paper is structured as follows. We start by elaborating on the mathematical similarity between F-DMAS and SLSC in Sec. 2.1, followed by detailed development that leads to the GSC beamformer. Sections 2.2, 2.3, and 2.4 describe the imaging metrics and simulation and experimental methods, respectively. Section 3 presents and discusses the results, and the conclusion is finally drawn in Sec. 4.

## 2 Materials and Methods

### 2.1 Generalized Beamformer Equation

First, the delay, and sum equation $y_{\mathrm{DAS}}$ is

$$y_{\mathrm{DAS}}(n)=\overset{N-1}{\underset{i=1}{\sum }}s_{i}(n),$$ (1)

where $s_{i}(n)$ is the delayed PA signal from the i’th transducer element at the n’th sample. Then, the delay, multiply, and sum beamformer equation, $y_{\mathrm{DMAS}}$ adds a multiplication step as a cross-correlator among different transducers:

$$y_{\mathrm{DMAS}}(n)=\overset{N-1}{\underset{i=1}{\sum }}\overset{N}{\underset{j=i+1}{\sum }}\mathrm{sign}[s_{i}(n)s_{j}(n)]\sqrt{\left|s_{i}(n)s_{j}(n)\right|},$$ (2)

where sign() is the signum function, and N is the number of elements in the transducer array. To extract the second harmonic components from the multiplication step, we filter the signals using a bandpass filter. We also rearrange terms in the summation by expressing sign() function and absolute value operator implicitly and adding the filter. In other words, using $\sqrt{x}=\frac{x}{\sqrt{x}}$ and convolving the expression with the filter, we can rewrite Eq. (2) as

$$y_{\mathrm{FDMAS}}(n)=h_{1}(n)*\overset{N-1}{\underset{i=1}{\sum }}\overset{N}{\underset{j=i+1}{\sum }}\frac{s_{i}(n)s_{j}(n)}{\sqrt[{4}]{s_{i}^{2}(n)s_{j}^{2}(n)}},$$ (3)

where $h_{1}(n)$ is the bandpass filter, and * denotes the convolution operation defined as

$$[f*h](n)=\overset{L}{\underset{\tau =-L}{\sum }}f(\tau )h(\tau -n).$$ (4)

For the SLSC beamformer, the normalized spatial coherence across the transducer array is used as the imaging contrast. The normalized spatial coherence at lag m (number of separation elements between the transducer elements) is defined as

$$R(m)=\frac{1}{N-m}\overset{N-m}{\underset{i=1}{\sum }}\frac{\sum_{n=n_{1}}^{n_{2}}s_{i}(n)s_{i+m}(n)}{\sqrt{\sum_{n=n_{1}}^{n_{2}}s_{i}^{2}(n)\sum_{n=n_{1}}^{n_{2}}s_{i+m}^{2}(n)}}.$$ (5)

The kernel size, $K=n_{2}-n_{1}$, is usually selected at one wavelength to strike a balance between the axial resolution and correlation stability.^18^ The SLSC beamformer sums R(m) in the first M lags to reach a suitable tradeoff between the lateral resolution and SNR:

$$RI=\overset{M}{\underset{m=1}{\sum }}R(m).$$ (6)

To make the similarity between FDMAS Eq. (2) and SLSC Eq. (5) more explicit, we further rewrite Eq. (3) as

$$y_{\mathrm{FDMAS}}(n)=\overset{N-1}{\underset{m=1}{\sum }}\overset{N-m}{\underset{i=1}{\sum }}h_{1}(n)*[s_{i}^{\prime }(n)s_{i+m}^{\prime }(n)],$$ (7)

where $s_{i}^{\prime }(n)=\frac{s_{i}(n)}{\sqrt[{4}]{\sum_{n=n_{1}}^{n_{1}}s_{i}^{2}(n)}}$. In other words, FDMAS adds all lags up to N−1. Similarly, we can re-express Eq. (6) as

$$RI=\overset{M}{\underset{m=1}{\sum }}\frac{1}{N-m}\overset{N-m}{\underset{i=1}{\sum }}\frac{\sum_{n=n_{1}}^{n_{2}}s_{i}(n)s_{i+m}(n)}{\sqrt{\sum_{n=n_{1}}^{n_{2}}s_{i}^{2}(n)\sum_{n=n_{1}}^{n_{2}}s_{i+m}^{2}(n)}}\;=\overset{M}{\underset{m=1}{\sum }}\frac{1}{N-m}\overset{N-m}{\underset{i=1}{\sum }}\overset{n_{2}}{\underset{n=n_{1}}{\sum }}\frac{s_{i}(n)}{\sqrt{\sum_{n=n_{1}}^{n_{2}}s_{i}^{2}(n)}}\frac{s_{i+m}(n)}{\sqrt{\sum_{n=n_{1}}^{n_{2}}s_{i+m}^{2}(n)}}\;=\overset{M}{\underset{m=1}{\sum }}\frac{1}{N-m}\overset{N-m}{\underset{i=1}{\sum }}\overset{n_{2}}{\underset{n=n_{1}}{\sum }}h_{2}(n)*[s_{i}^{\prime }(n)s_{i+m}^{\prime }(n)],$$ (8)

where $s_{i}^{\prime }(n)=\frac{s_{i}(n)}{\sqrt[{2}]{\sum_{n=n_{1}}^{n_{2}}s_{i}^{2}(n)}}$ and $h_{2}(n)=[1,1,\ldots ,1]$, which describes a low-pass filter. The resemblance of SLSC and FDMAS is now evident, and we can generalize them to a mathematical equation that encompasses both beamformers:

$$y(n)=\overset{M}{\underset{m=1}{\sum }}w(m)\overset{N-m}{\underset{i=1}{\sum }}h(n)*\{g[s_{i}(n)]g[s_{i+m}(n)]\},$$ (9)

where w(m) represents a weight function, h(n) is a filter, and $g[s_{i}(n)]$ is given as

$$g[s_{i}(n)]=\;\{\begin{array}{ll} \frac{s_{i}(n)}{\sqrt[{4}]{\sum_{n=n_{1}}^{n_{1}}s_{i}^{2}(n)}}, & \mathrm{FDMAS}\\ \frac{s_{i}(n)}{\sqrt[{2}]{\sum_{n=n_{1}}^{n_{2}}s_{i}^{2}(n)}}, & \mathrm{SLSC} \end{array}.$$

Function $g[s_{i}(n)]$ highlights two key differences between the two beamformers. First, FDMAS preserves the signal magnitude by employing a weaker quasi-normalization through fourth root. On the other hand, SLSC is more robust against noise using a larger kernel for coherence evaluation, resulting in a better CNR. This implies that FDMAS’ inferior CNR is due to the fact that it uses only one point in the kernel for computing coherence.^30^ To prove the latter, we consider the PA signals received by a transducer element as a sum of signal $f_{i}(n)$ and uncorrelated noise $\phi_{i}(n)$: $s_{i}(n)=f_{i}(n)+\phi_{i}(n)$, with both being zero-mean. We also assume the noises of different transducer elements are uncorrelated but have the same variance $\sigma^{2}$. It is worth noting that we only considered random system noises.^31^ Then,

$$\overset{n_{2}}{\underset{n=n_{1}}{\sum }}s_{i}^{2}(n)\overset{n_{2}}{\underset{n=n_{1}}{\sum }}s_{i+m}^{2}(n)≅[\overset{n_{2}}{\underset{n=n_{1}}{\sum }}f_{i}^{2}(n)+\overset{n_{2}}{\underset{n=n_{1}}{\sum }}\sigma_{i}^{2}(n)]\cdot [\overset{n_{2}}{\underset{n=n_{1}}{\sum }}f_{i+m}^{2}(n)+\overset{n_{2}}{\underset{n=n_{1}}{\sum }}\sigma_{i+m}^{2}(n)]\;≅[\overset{n_{2}}{\underset{n=n_{1}}{\sum }}[f_{i}^{2}(n)+\sigma^{2}]+\overset{n_{2}}{\underset{n=n_{1}}{\sum }}\varphi_{i}(n)]\cdot [\overset{n_{2}}{\underset{n=n_{1}}{\sum }}[f_{i+m}^{2}(n)+\sigma^{2}]+\overset{n_{2}}{\underset{n=n_{1}}{\sum }}\varphi_{i+m}(n)],$$ (10)

where $\overset{n_{2}}{\underset{n=n_{1}}{\sum }}[f_{i}(n)\sigma_{i}(n)]≅0$ has been used since the noise is uncorrelated to the signal. $\phi_{i}^{2}(n)$ is decomposed into its mean variance $\sigma^{2}$ and a zero-mean component $\varphi_{i}(n)$, which causes the coherence value to fluctuate even though the noise power $\sigma^{2}$ remains the same. A larger kernel K effectively reduces the effect of $\varphi_{i}(n)$ relative to $\left[f_{i+m}^{2}(n)+\sigma^{2}\right]$ by a factor of $\sqrt{K}$, leading to a statistically more robust evaluation of spatial coherence in SLSC.^32^

The filter h(n) in Eq. (8) is utilized in FDMAS and SLSC differently. The FDMAS implements a bandpass filter that selects the second harmonic component. In contrast, SLSC uses an integration-based low-pass filter. Theoretically, if the chosen kernel size is large enough so that the PA signals inside the kernel are zero-mean, both filters would yield the same spatial coherence. Otherwise, using the second harmonic components is likely to underestimate the coherence because short kernels can prevent second harmonic components to fully develop as opposed to the DC component; the kernel length must cover at least one period waveform of the transducer’s center frequency. Nevertheless, the second harmonic component and hence the bandpass filter can be advantageous in certain applications. For instance, in power Doppler Ultrasound, a higher central frequency is typically desired,^33^ which, therefore, can benefit from the second harmonic components that can be extracted by a bandpass filter in GSC/FDMAS.

Lastly, the weight function w(n) assigns scores to coherence at different lags. In SLSC, shown in Eq. (6), the scores are assigned uniformly: the N−m transducer signal pairs’ coherence at lag m is divided by N−m. In contrast, F-DMAS sums the N−m pairs’ quasi-normalization signals at lag m without dividing by N−m, which effectively assigns to the coherence a weight of N−m, promoting the contributions from smaller lags m. As the coherence is generally smaller at larger lags, coherences at larger lags contribute less to the overall imaging contrast. Therefore, a non-uniform weight function is beneficial for optimizing the imaging contrast.^24^^–^^26^

Motivated by the considerations above, we present GSC beamformer equation:

$$y_{\mathrm{GSC}}(n)=w(n)\overset{M}{\underset{m=1}{\sum }}\overset{N-m}{\underset{i=1}{\sum }}h(n)*[\frac{s_{i}(n)}{\sqrt[{4}]{\sum_{n=n_{1}}^{n_{2}}s_{i}^{2}(n)}}\frac{s_{i+m}(n)}{\sqrt[{4}]{\sum_{n=n_{1}}^{n_{2}}s_{i+m}^{2}(n)}}],$$ (11)

where $g(n)=\frac{s_{i}(n)}{\sqrt[{4}]{\sum_{n=n_{1}}^{n_{2}}s_{i}^{2}(n)}}$ preserves signal magnitude, h(n) is a low-pass filter that extracts DC components. The lags are limited to the first M values by selecting w(n)=1 for m<M and 0 otherwise to preferentially boost the contributions from small lags. By employing a non-uniform weight function w(n) and a quasi-normalization function with finite kernel, the proposed GSC beamformer goes beyond combining the merits of F-DMAS and SLSC: it not only preserves the signal strength but also provides an even higher contrast and noise robustness than F-DMAS and SLSC.

### 2.2 Imaging Metrics

Imaging performance is characterized by three different metrics: contrast (C), SNR, and generalized CNR (gCNR), which are defined respectively as^19^

$$C=20\;\log\;\frac{S_{i}}{S_{o}}.$$ (12)

$$\mathrm{SNR}=20\;\log\;\frac{\left|S_{i}\right|}{\sqrt{\sigma_{o}^{2}}}.$$ (13)

$$\mathrm{gCNR}=1-\overset{N-1}{\underset{k=0}{\sum }}\min\{h_{i}(x_{k}),h_{o}(x_{k})\}.$$ (14)

Here, $S_{i}$ and $S_{o}$ are the mean brightness values of the image inside and outside the target, respectively, and $\sigma_{o}^{2}$ refers to brightness variance outside the target, $h_{i}$ and $h_{o}$ are the associated histograms inside and outside the target, respectively, and $x_{k}$ refers to the index of the bin with a total of N bins. Shall be noted that gCNR is a relatively new imaging metric, which measures lesion detectability within a range from 0 to 1, where 1 is maximum detectability.^34^^,^^35^ As opposed to traditional CNR calculations, gCNR provides a more linear relationship between imaging metrics and image quality.

### 2.3 Simulations

We first conducted a simulation study using the K-wave toolbox in MATLAB.^36^ The simulation was initiated in a two-dimensional grid containing 512×512 points. Total grid size was 20×20  mm. To maintain a balance between numerical model stability and computational speed, we define Courant–Friedrichs–Lewy (CFL) as $CFL=c_{0}\Delta t/\Delta x$, where $c_{0}$ is the speed of sound in tissue (1500  m/s), Δt is the time step, and Δx is the step size between the point grids, and CFL=0.3. The simulated transducer has the same parameters as the custom transducer probe used in experiments—a linear array with a central frequency at 2.5 MHz and an 80% fractional bandwidth. The transducer parameters are shown in Table 1.

**Table 1:** Transducer parameters.

Parameter	Value
Number of elements	128
Pitch	0.67 mm
Sampling frequency	14.925 MHz
Center frequency	2.5 MHz
Fractional bandwidth	80%

We performed four different simulations. First, to calculate the point spread function (PSF) of the four considered beamformers (DAS, FDMAS, SLSC, and GSC), point sources are located at 10 mm away from the center of the array. To simulate the noise effect, we normalized the radio-frequency data against the globally maximum signal amplitude across all channels, followed by bandpass filtering the signal to simulate finite bandwidth of transducers and lastly adding white noises. Second, we evaluated the beamformers’ capability in preserving the signal magnitude by assigning three point sources separated by a few millimeters at the same depth with different absorption weights. Then, we confirmed the relationship between the selected lag in GSC beamforming and imaging metrics, and lateral resolution. Lastly, to compare the performance of these beamformers in a realistic scenario, we reconstructed a vessel phantom at five different noise levels.

### 2.4 Experiments

The experimental setup consists of a water tank with an opening partially sealed with a fluorinated ethylene propylene (FEP) plastic film (McMASTER-Carr), used as an imaging window. Pulse-echo measurements were used to verify that there was no acoustic attenuation through 50-μm-thick FEP film. Light transmission was found to be >97%. A 20-cm stroke translation stage (McMASTER-Carr) mounted on an optical breadboard was used to ensure linear scanning. The ultrasound transducer is a custom made, 128-element linear array with curved elements to facilitate acoustic focusing without using a lens, with an element pitch of 0.67 mm and a central frequency of 2.25 MHz (Imasonics, Inc.) connected to a Vantage 256 data acquisition system (Verasonic, Inc.). A 10 nanosecond pulse Nd:YAG laser with 10-Hz pulse repetition rate at 1064-nm wavelength output (Continuum, SL III) was used as the excitation source. The laser was coupled to one of the bifurcated line output fibers that has an input diameter of 1 cm and an output length of 8 cm (Schott). Light delivery and acoustic detection were synchronized with trigger output from the laser. A 3D printed holder was used to fix two dichroic mirrors (TECHSPEC hot mirror and cold mirror, Edmund Optics Inc.), which were placed at 45-deg angles to the transducer and fiber. The reflection through the hot mirror is 90% at 45-deg incidence for 1064-nm light. The cold mirror transmits 97% for the same angle of incidence and wavelength. The light illumination is co-planar with acoustic detection in this design.^37^^–^^39^

**Fig. 1:**
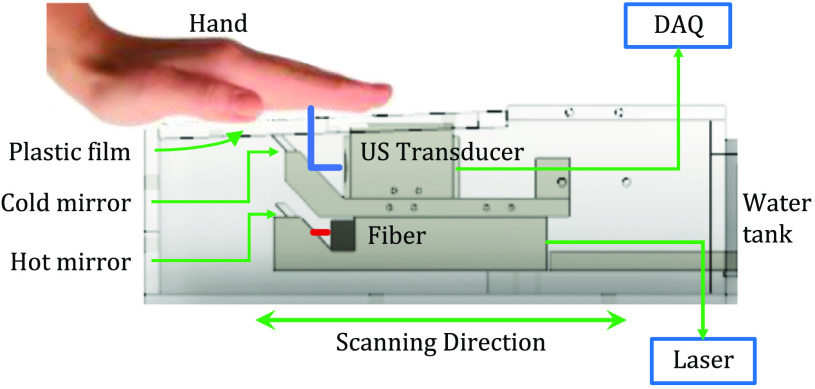
A schematic drawing of the palm imaging setup.

All human procedures were performed in compliance with the University at Buffalo IRB protocol. All volunteers were enrolled after consent documents were signed. During imaging, the palm was placed on the plastic film with ultrasound gel as the coupling medium (Parker Laboratories, Inc.). The transducer-fiber bundle set (scan head) fixed in the 3D printed holder was immersed into the water tank as shown in Fig. 1. Energy irradiated on the palm was measured as $21\;\mathrm{mJ}/{\mathrm{cm}}^{2}$, which is well below the ANSI safety limit of $100\;\mathrm{mJ}/{\mathrm{cm}}^{2}$ for 1064-nm light.^40^ The palm was scanned linearly with a step size of 0.1-mm/laser pulse. With this setup, we have an imaging window of 20  cm×10  cm.

## 3 Results

### 3.1 Simulation Results

**Fig. 2:**
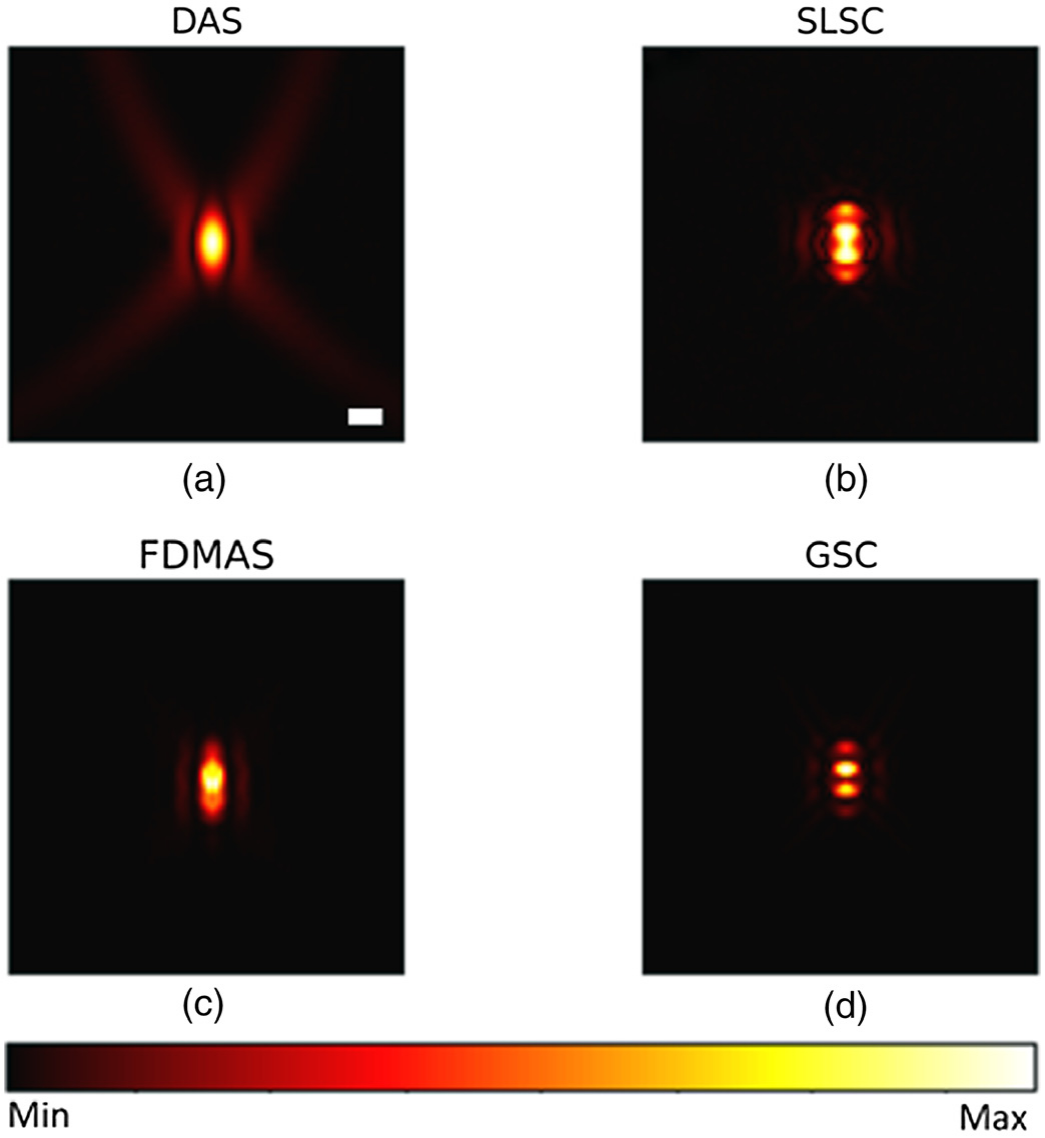
Point source noise-free reconstruction using (a) DAS; (b) SLSC; (c) FDMAS; (d) GSC. Scale bar: 1 mm.

**Fig. 3:**
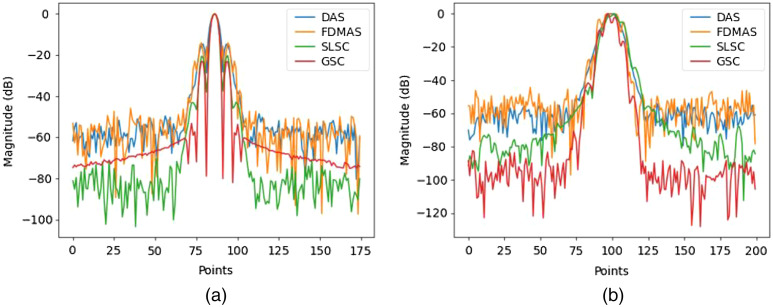
Noise-free point reconstruction line profiles: (a) lateral line profile and (b) axial line profile.

Figures 2(a)–2(d) shows the PSFs of four different beamformers: DAS, SLSC, FDMAS, and GSC, respectively. In SLSC and GSC reconstruction, the M lag was selected as 0.7 of the transducer aperture to strike a balance between the lateral resolution and other imaging metrics, and the kernel size was set to one wavelength, these values’ selections will be further discussed. Such PSFs represent the ideal case scenario where the sensor data has −40  dB noise amplitude. Normalized lateral line profiles of the PSFs for each beamforming technique are shown in Fig. 3(a). The lateral resolution was measured for each technique by calculating the full-width half maximum of line profiles with FDMAS being the lowest, 152  μm, followed by GSC, 158  μm, SLSC 181  μm, and DAS, 193  μm. GSC shows lowest side lobes. Figure 3(b) shows normalized axial profile, where GSC shows highest contrast.

**Fig. 4:**
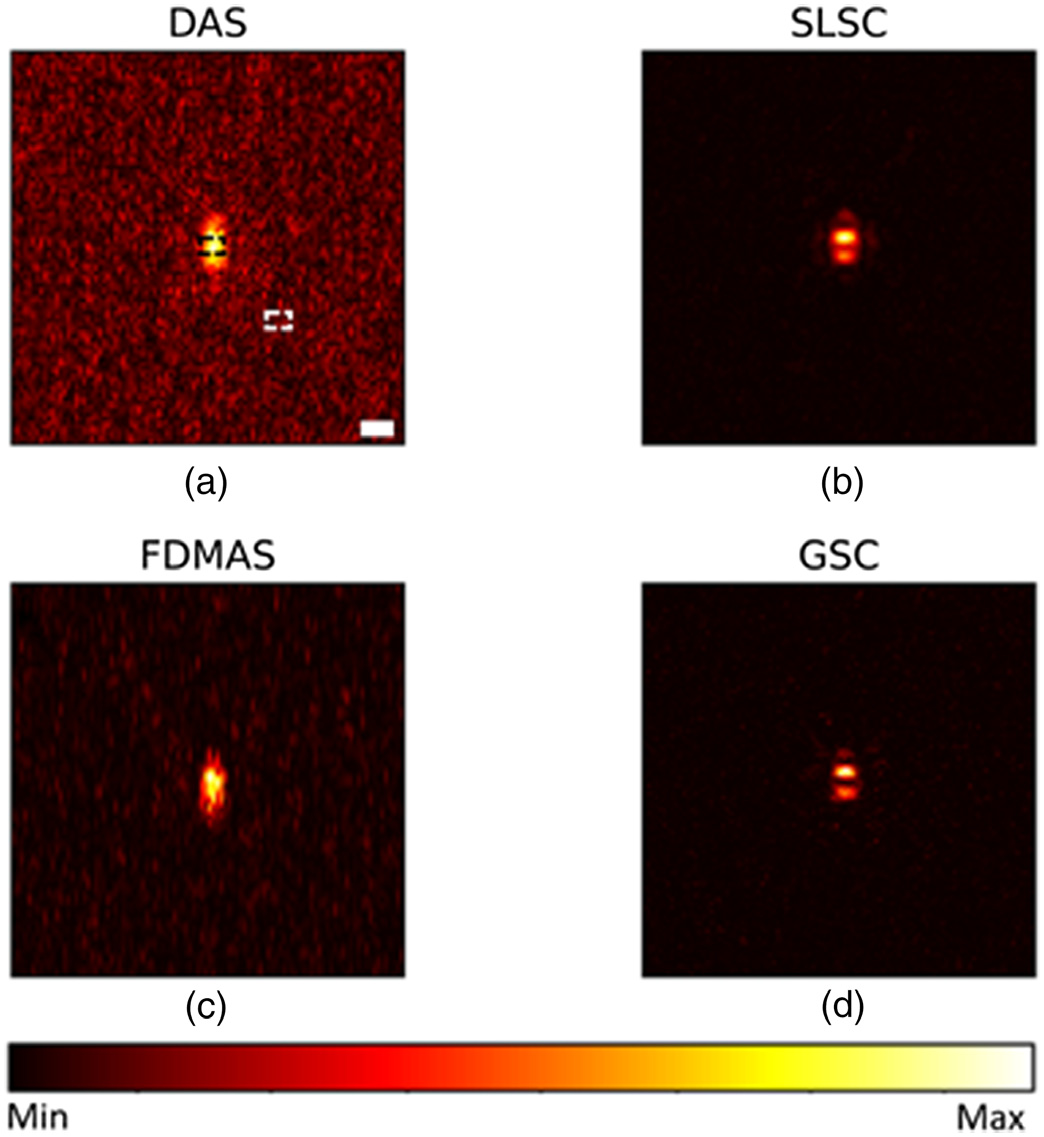
Point source reconstruction under −12  dB noise level using. (a) DAS; (b) SLSC; (c) FDMAS; (d) GSC. Scale bar: 1 mm.

**Fig. 5:**
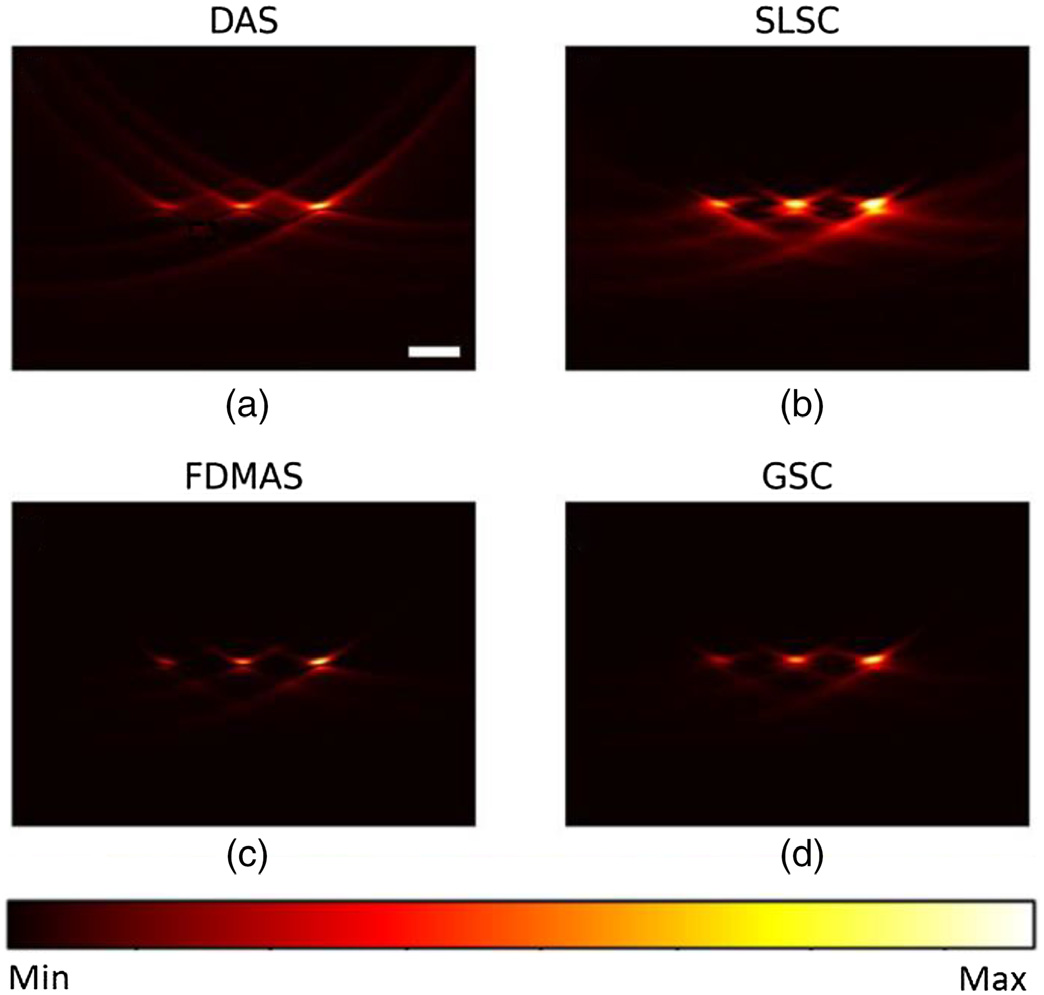
Absorption reconstruction for point sources with different weights using (a) DAS; (b) SLSC; (c) FDMAS; (d) GSC. Scale bar: 1 mm.

**Fig. 6:**
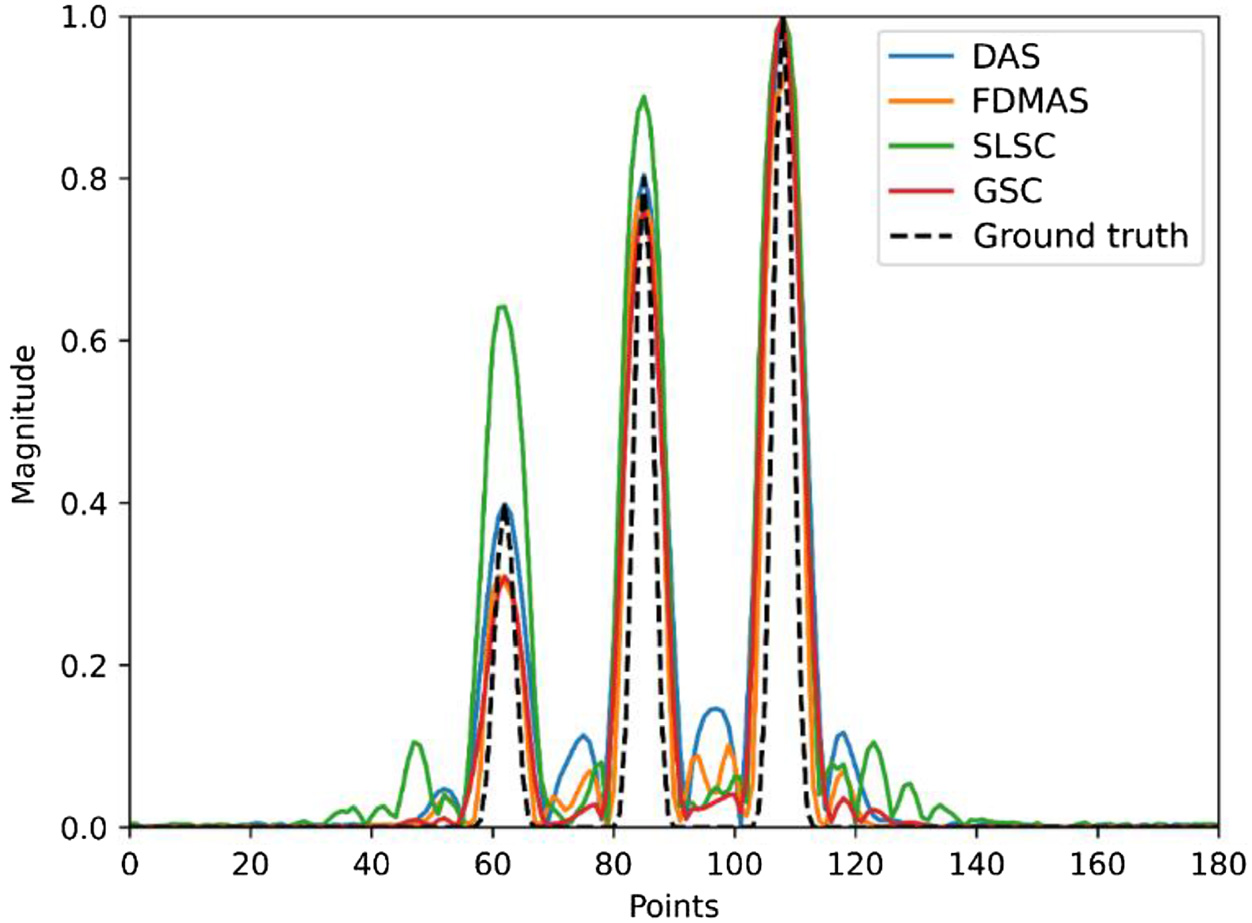
Lateral profile of weighted point sources in Fig. 5 showing SLSC magnitude loss.

Figure 4 shows the PSF reconstructed with a −12  dB noise in sensor data. Overall, SLSC and GSC show a similar lateral resolution values as in Fig. 2 and improved imaging performance compared with DAS and FDMAS owing to their robustness to noise. Particularly, GSC shows the highest contrast, 41.2 dB, and SNR, 41.8 dB, followed by SLSC with 40.6 and 40.6 dB, FDMAS with 24.8 and 24.8 dB, and DAS 14.8 and 21.1 dB, respectively. Figures were normalized for quantitative analysis. Although the image performance difference between SLSC and GSC is just a few decibels, SLSC cannot preserve the relative signal magnitude. To illustrate this fact, we reconstructed the three point sources with an absorption coefficient of 0.4, 0.8, and 1 using different beamformers in Fig. 5 and compared the line profiles of reconstructed absorption coefficients from all beamformers, along with ground truth, in Fig. 6. The signal magnitude difference between SLSC and other beamformers is notable: while other techniques manage to recover the relative absorption magnitude, SLSC loses the signal magnitude due to its normalization operation.

**Fig. 7:**
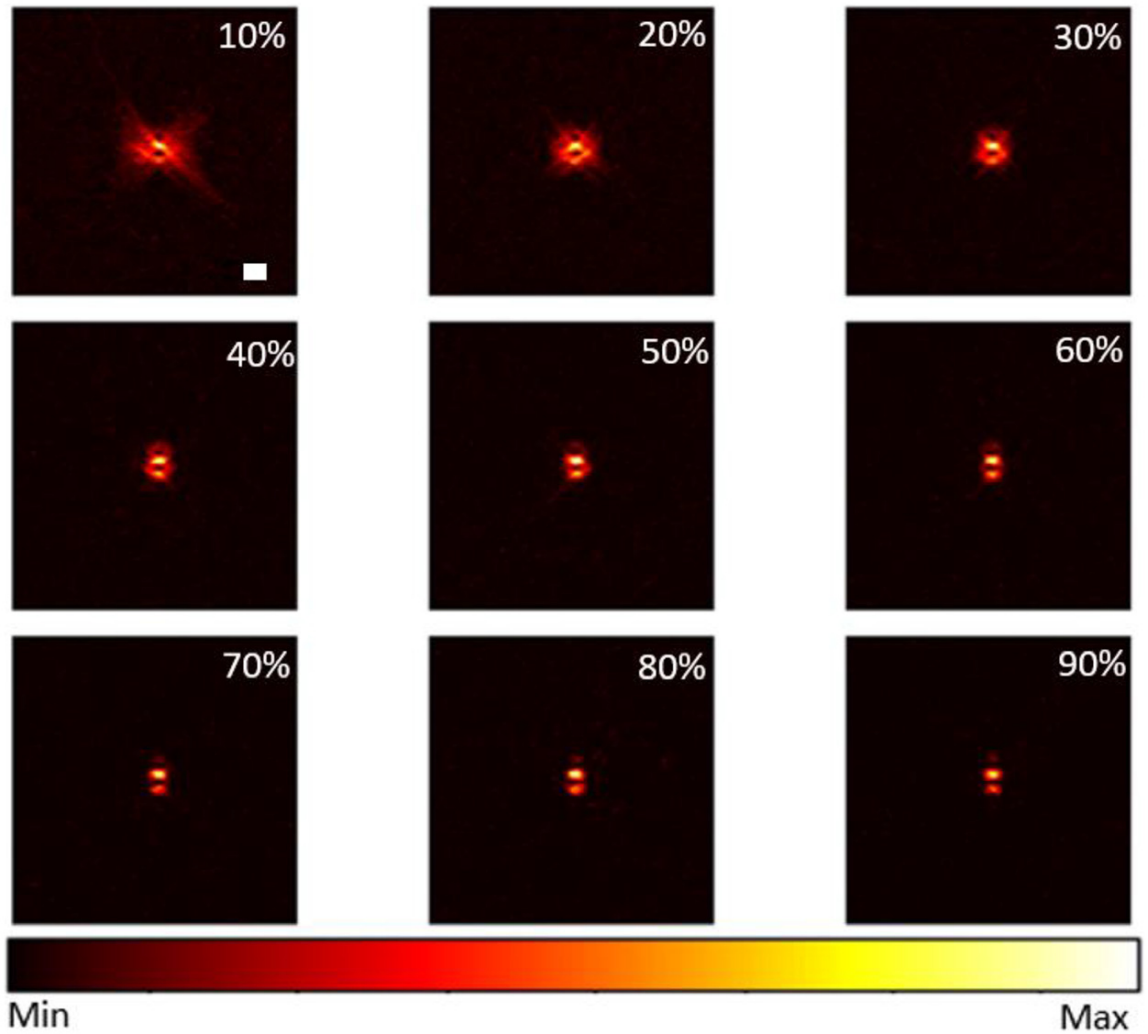
GSC’s point source reconstruction with different maximum lags. Scale bar: 1 mm.

Figure 7 shows the GSC PSF reconstruction of the same point source simulated in Fig. 4 with changing lag, from 10% to 90%, respectively. The lateral resolution is generally improved as the lag increases. However, as shown in Table 2, imaging metrics do not follow the same relationship with respect to lag. Both contrast and SNR reach highest values at 70% lag while lesion detectability remains roughly constant. These results result in agreement with VCZ photoacoustic theory stated in Ref. 15.^15^

**Table 2:** GSC’s imaging metrics with different maximum lag.

Lag (%)	C (dB)	SNR (dB)	gCNR (dB)
10	28.94	34.96	0.8
20	30.06	35.02	0.8
30	32.76	38.79	0.9
40	33.44	39.46	0.9
60	40.74	40.74	0.9
70	40.66	40.66	0.9
80	40.50	40.48	0.9
90	37.08	39.47	0.9

**Fig. 8:**
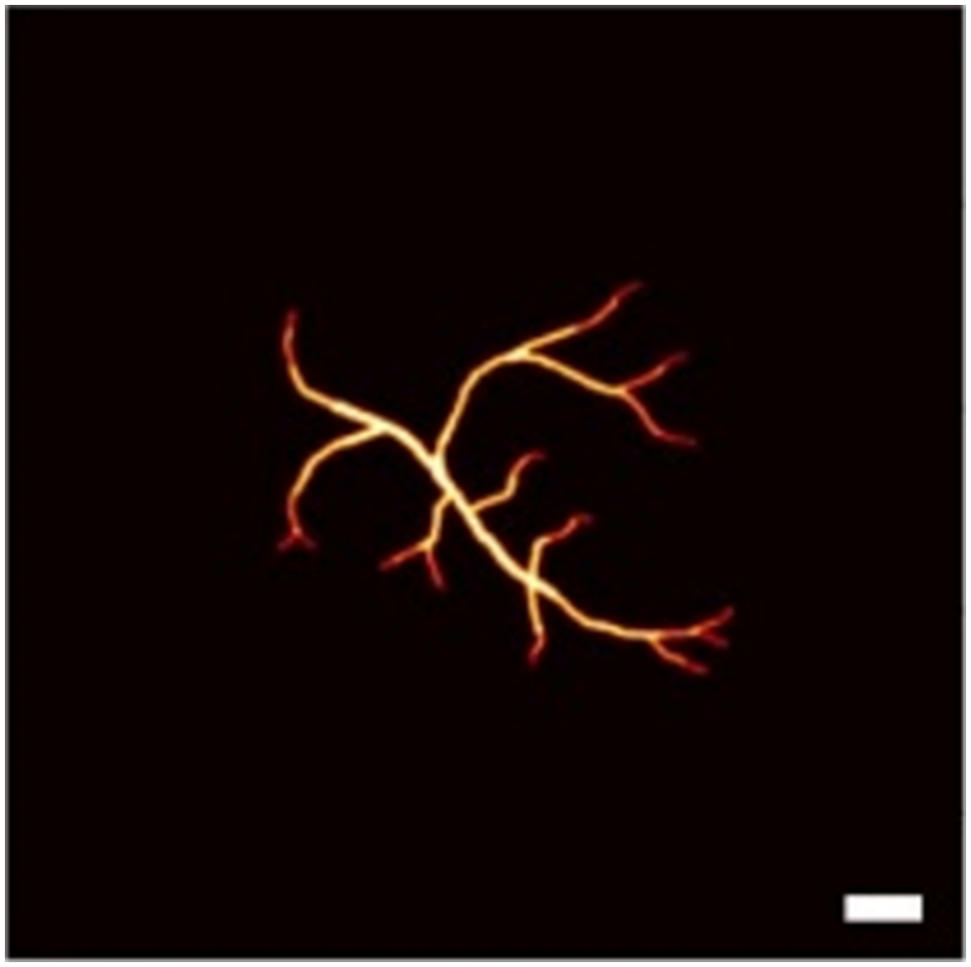
Ground truth consisting of vessel phantom. Scale bar: 1 mm.

**Fig. 9:**
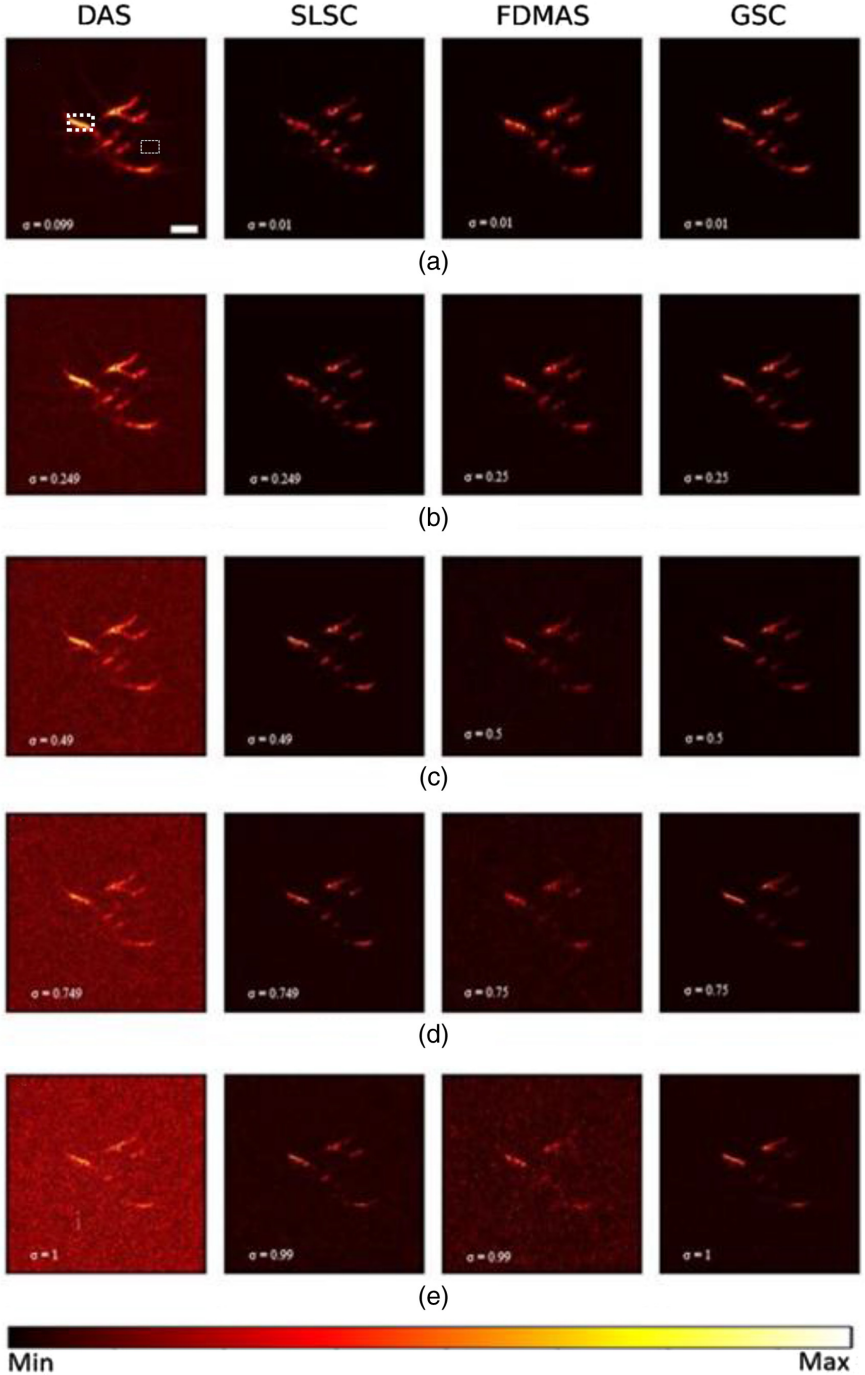
Vessel phantom reconstruction at five different noise levels: (a) −20; (b) −12; (c) −10; (d) −5; (e) −1  dB. The σ indicates noise standard deviation for each reconstruction. Scale bar: 1 mm.

To further compare the performance of all the considered beamformers, we simulated a vessel phantom (Fig. 8) with five different noise levels. The M   lag was chosen as 30% of the transducer aperture with the kernel size being one wavelength in SLSC and GSC. Figure 9 shows reconstruction results at different noise levels: −20, −12, −10, −5  dB, and −1  dB from Figs. 9(a)–9(e), respectively. Because the transducer’s geometry is linear, it suffers from a limited-view problem: structures orthogonal to the transducer array cannot be well reconstructed. At −20  dB noise in Fig. 9(a), all techniques yield accurate reconstruction of the vessel phantom. Compared with DAS, the resolution improvement achieved by FDMAS, SLSC, and GSC follows the same relationship as in point source simulations, due to FDMAS’s central frequency and SLSC and GSC’s noise robustness. At higher noise levels (−12 and −10  dB), the image quality of all beamformers decreases, with DAS being more sensitive to noise and followed by FDMAS. The reconstructed vessel is barely observable by DAS and FDMAS with −5-dB noise level [Fig. 9(d)] while SLSC and GSC can still reconstruct it. Finally, at −1  dB noise level [Fig. 9(e)], DAS and FDMAS cannot reconstruct the main features of the vessel, and SLSC has reduced image quality compared to GSC. It is worth noting that at −10 and −5  dB noise levels, SLSC tends to make weaker regions to appear brighter in the image due to magnitude information loss.

**Fig. 10:**
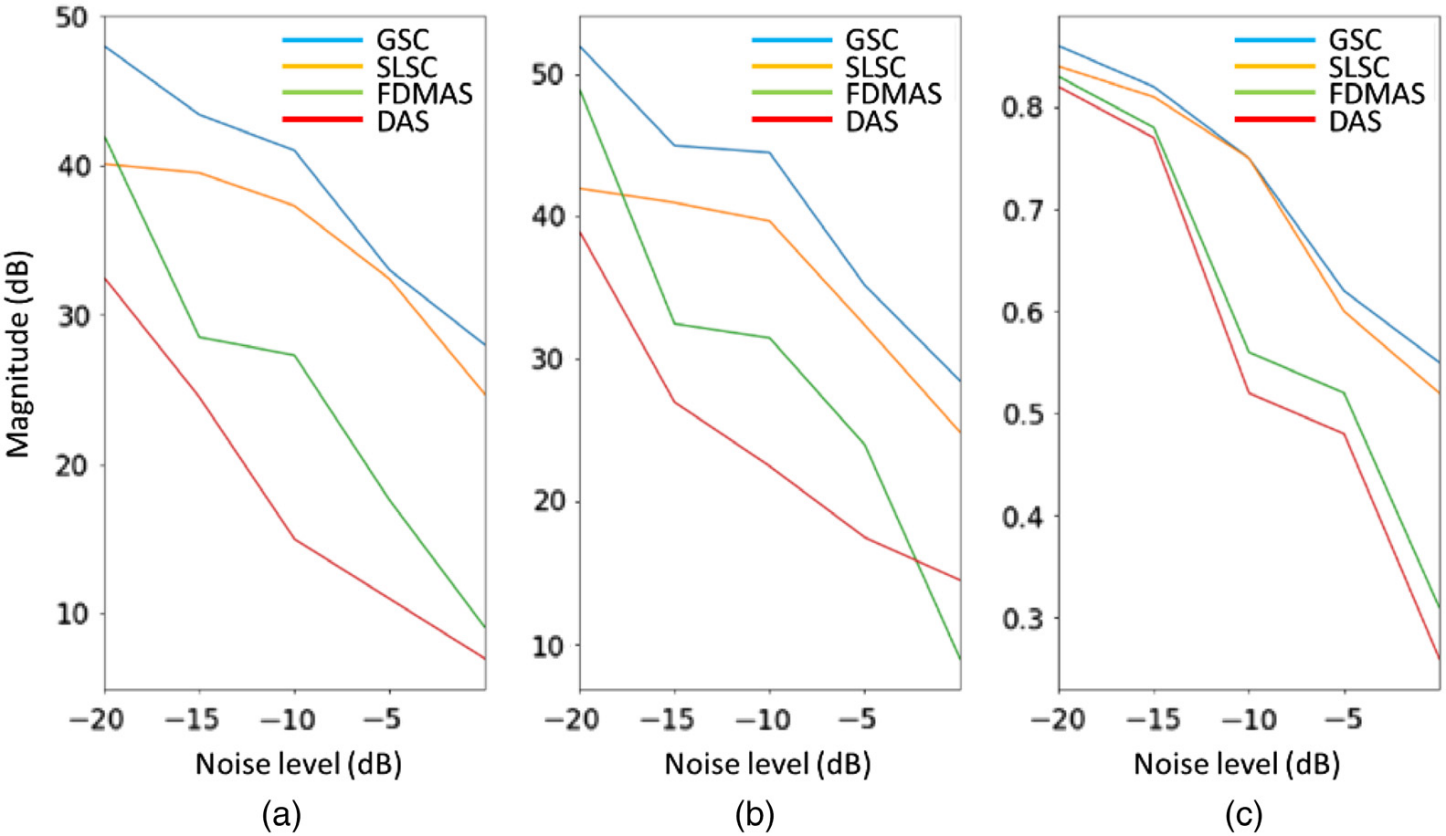
Imaging metrics (a) Contrast; (b) SNR; (c) gCNR for different noise levels in Fig. 9.

Figure 10 compares the reconstructed images from Fig. 9 quantitatively. In terms of contrast, GSC and SLSC show an approximately same decreasing trend with increased noise levels, whereas DAS and FDMAS exhibit a higher negative gradient as they are less robust to noise. At the −10-dB noise level, the contrast in GSC is 4, 14, and 26 dB higher than those in SLSC, FDMAS, and DAS, respectively. GSC achieved a consistently better contrast than DAS, SLSC, and FDMAS at all noise levels.

Regarding the SNR, GSC consistently shows the highest value, whereas DAS and FDMAS are less robust to noise. At lower noise levels (−20 and −15  dB), the differences between GSC and SLSC, FDMAS, and DAS are 9, 3, and 17 dB, respectively. And at the highest noise level, these differences are 5, 22, and 15 dB, respectively.

The gCNR shows a similar dependence on noise as with contrast and SNR. All beamforming techniques show between 0.8 and 0.9 detectability with a −20  dB noise level. At the second noise level, we can see two groups clearly differentiate: on the one hand, GSC and SLSC, and on the other hand, FDMAS and DAS. Again, noise robustness plays a key role. At −5 and −2  dB, GSC shows the highest lesion detectability with values above 0.6 and 0.55, respectively, followed by SLSC with 0.2 difference. At the highest noise level, DAS and FDMAS cannot reconstruct the vessel with high fidelity, and their gCNR are 0.27 and 0.32, respectively.

**Fig. 11:**
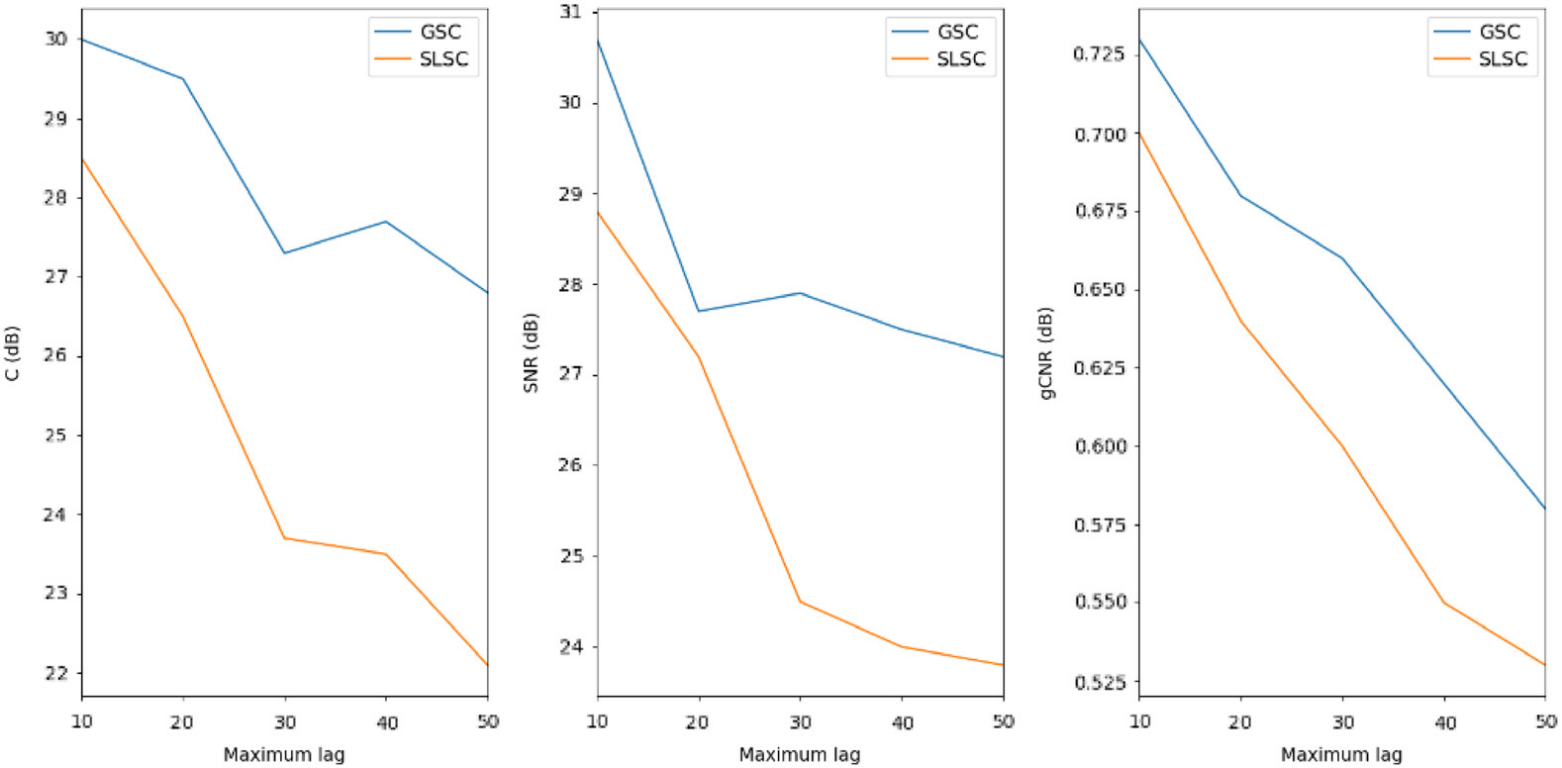
Contrast and SNR in SLSC and GSC with different maximum lag selection in vessel phantom reconstruction.

It is worth noting that while GSC overperforms SLSC using the same parameters, the magnitude difference in imaging metric from other beamformers depends on the selected parameters, particularly M lag. Figure 11 shows SLSC and GSC’s contrast, SNR, and gCNR magnitude variability as a function of lag for a −10  dB noise level in the vessel phantom reconstruction: as the lag increases, there is an overall descending gradient, indicating the aforementioned tradeoff between imaging metrics and the lateral resolution.

### 3.2 Experimental Results

**Fig. 12:**
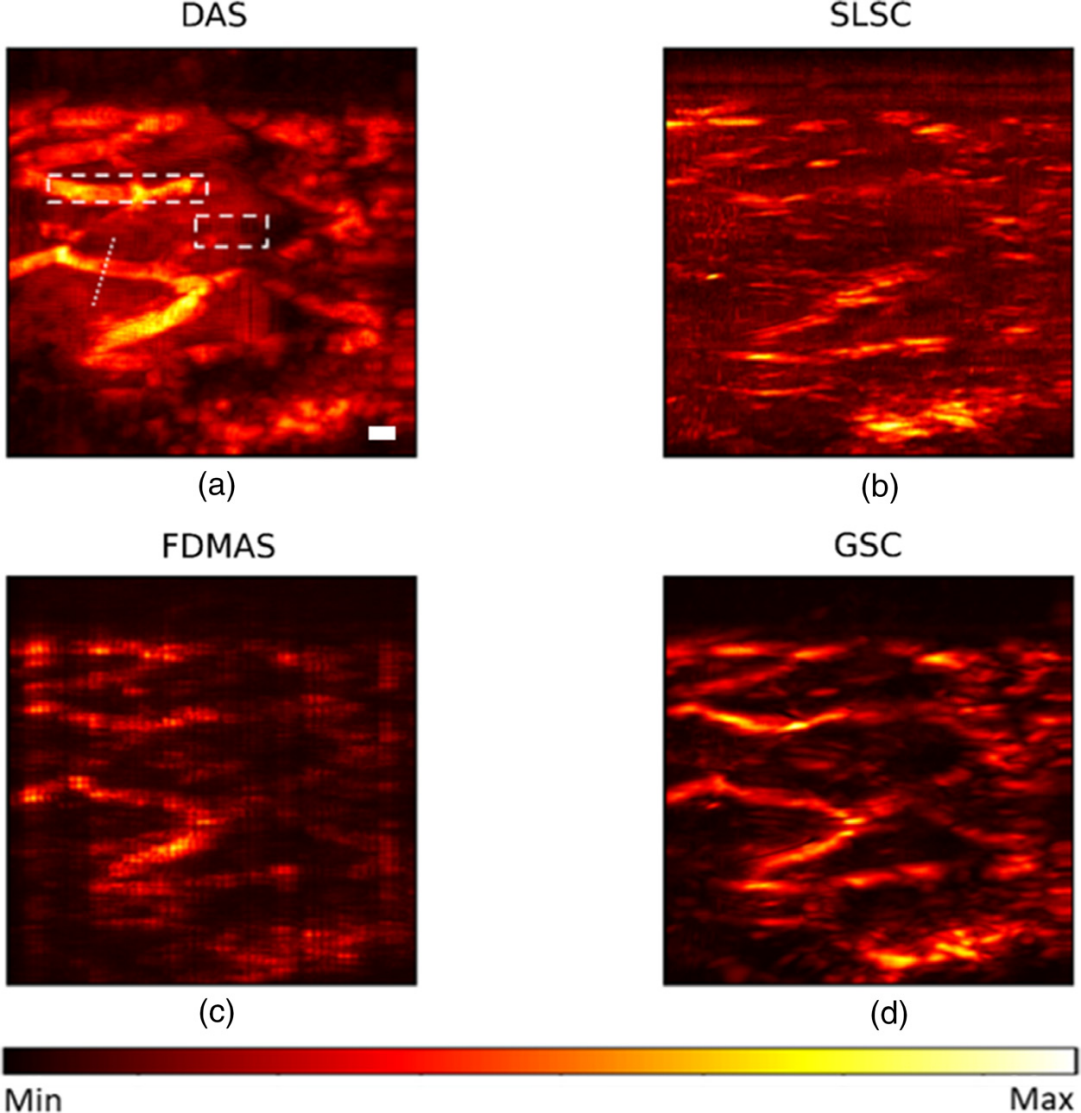
Palm experimental reconstruction (a) DAS; (b) SLSC; (c) FDMAS; (d) GSC. Scalebar: 2 mm.

After 3D imaging experimental data acquisition, we performed 3D beamforming in a slice-by-slice manner. Next, we took the maximum amplitude projection of the reconstructed 3D image along the depth direction. For SLSC and GSC, $M_{lag}$ was selected as 30% of the transducer aperture, respectively, and the kernel size is one wavelength. The reconstruction results are shown in Fig. 12. DAS palm’s reconstruction has a high background noise, and surprisingly, SLSC has some noise artifacts that we believe are due to slicing during data acquisition. FDMAS suffers from a reduced contrast to noise ratio, as observed previously. GSC shows the noticeable improved image quality compared with other beamformers: it has the lowest background noise and the highest contrast. In terms of the lateral resolution, FDMAS and GSC improve with respect to DAS and SLSC. A line profile of an arbitrary vessel shown in Fig. 11 was taken with ∼1.7  mm for FDMAS and GSC, and ∼2  mm in DAS. The resolution was not measured in SLSC due to aforementioned artifacts.

We summarized the imaging metrics for experimental reconstructions in Table 3. GSC shows an 8, 10, and 13 dB increase in contrast compared with FDMAS, DAS, and SLSC, respectively. GSC also has the highest gCNR followed by FDMAS with 0.05 difference, DAS, and SLSC. SLSC’s gCNR is dominated by the artifacts, which also make its SNR is the lowest. In terms of SNR, GSC is at least 3 dB higher than all other beamformers. Overall, the experimental results corroborate our simulation finding that GSC outperforms DAS, FDMAS, and SLSC in all imaging metrics.

**Table 3:** Imaging metrics in experimental palm results.

	Contrast (dB)	SNR (dB)	gCNR
DAS	8.94	25.2	0.73
SLSC	6.67	17.3	0.54
FDMAS	11.29	20.9	0.81
GSC	19.63	28.0	0.86

## 4 Discussion

As opposed to FDMAS, where the improved lateral resolution in FDMAS both simulated and experimental results is attributed to its doubled central frequency of the signal, which halves the wavelength, in GSC it is mainly due to noise robustness. Although, in this paper, GSC does not utilize a bandpass filter, it could be also used to further improve the resolution in applications where frequency is critical. Such case could be elasticity imaging, where the low-frequency components worsen lateral resolution.^41^

As with SLSC, GSC’s lag selection is empirical. For instance, in cases where noise levels are relatively low it would be desirable to maximize lag to improve lateral resolution other imaging metrics should not be highly affected. However, in real case scenarios, noise levels are much higher, especially in deeper tissue locations where light fluence is low and there is low acoustic signal, the lag cannot be increased. Otherwise, imaging metrics will be considerably degraded. Using relatively small M lag values optimizes the imaging contrast, whereas larger M lag values yield better lateral resolution because the effective aperture is increased. Regarding the kernel selection, the rule of thumb is to select a value close to acoustic wavelength to strike a balance between the axial resolution and correlation stability. Overall, GSC beamformer for PACT goes beyond combining the strengths of FDMAS and SLSC. As in FDMAS, it preserves the signal strength. Like SLSC, it is more robust to the noise using a finite kernel for coherence evaluation. More importantly, it achieved high quality imaging performances in contrast, CNR and SNR. GSC’s lateral resolution improvement difference remains depending on the filter choice and the selected M lag.

Although there exist previous modifications and combinations of FDMAS and SLSC, GSC combines the best from both original techniques. SLSC’s modifications include M-weighted SLSC^24^ and locally weighted-SLSC^25^^,^^26^ with non-uniform weighting as in FDMAS but, as opposed to GSC, the relative signal magnitude is not preserved preventing use in quantitative applications. There have also been FDMAS modifications, such as in Ref. 27, where a coherence factor has been implemented to increase resolution and SNR. However, in this technique, the kernel is still minimum, affecting CNR. Combinations of both algorithms such as SL-FDMAS^28^ still have the same drawbacks as conventional SLSC. GSC is a more versatile and general algorithm that can span more applications.

GSC has a similar computational cost as SLSC and higher than FDMAS and DAS. Since it utilizes a finite kernel for computing coherence values rather than a single point as in FDMAS, its computational cost is a few times higher depending on the kernel size. Hence, to achieve real-time imaging using GSC, FDMAS, or SLSC, it is necessary to employ parallel beamforming using a graphical processing unit (GPU).^42^ For a 512×512 reconstruction grid, our achieved frame rate is ∼10  Hz using Nvidia RTX2080Ti GPU.

To conclude, we mathematically generalized FDMAS and SLSC beamformers into a single beamformer equation, the GSC. GSC goes beyond combining the merits of SLSC and FDMAS: it preserves the signal strength and shows the high quality images for highly noisy measurements. GSC’s enhanced performance is particularly useful for deep PACT that suffers from higher noises due to a low light fluence.
